# Successful treatment with azithromycin and rifampicin of penicillin and cephalosporin insensitive pneumococcal osteomyelitis in a child with HIV infection: a case report

**DOI:** 10.1186/1757-1626-1-283

**Published:** 2008-10-29

**Authors:** Andrew I Riordan, Shazia Adalat, Clive Graham

**Affiliations:** 1 Department of Child Health, Birmingham Heartlands Hospital, UK; 2 Department of Microbiology, Birmingham Children's Hospital, Birmingham, UK; 3 Haematology Treatment Centre, Royal Liverpool Children's Hospital, Eaton Road, Liverpool, L12 2AP, UK

## Abstract

Pneumococcal infection is common in children with HIV infection, but osteomyelits is unusual. The best treatment for bone and joint infection due to antibiotic resistant pneumococci is not known, especially in immunocompromised children.

A 6 month old girl, infected with HIV by mother to child transmission, had recently started combination antiretroviral therapy (cART). She presented with osteomyelitis of the left radius confirmed on bone scan. Blood cultures grew *Streptococcus pneumoniae *9S resistant to penicillin, with reduced susceptibility to ceftriaxone.

Osteomyelitis was treated with parenteral teicoplanin, oral rifampicin and azithromycin. After two weeks of treatment she developed rash and fever. These were thought to be a drug eruption and resolved when teicoplanin was stopped. She completed a 3 month course of rifampicin and azithromycin and continued on cART. She has normal function of her left wrist 18 months after treatment. She remains on her original cART regimen with an undetectable viral load and normal CD4 count (34%; 1398 × 10^6^/l).

The combination of rifampicin and azithromycin was well tolerated, simple to administer and effective. This combination deserves further study in bone and joint infection caused by antibiotic resistant Gram positive bacteria.

## Introduction

*Streptococcus pneumoniae *is the commonest pathogen causing bacteraemia in children infected with the human immunodeficiency virus (HIV). However osteomyelitis is reported infrequently, and osteomyelitis due to penicillin-resistant *S. pneumoniae *has not been reported previously in this patient group [1].

We report successful treatment of penicillin and cephalosporin non-susceptible pneumococcal osteomyelitis in a child with HIV, using rifampicin and azithromycin.

## Case presentation

A 6 month old HIV positive black African girl presented with a two week history of intermittent fever. Her mother had been found to be HIV positive when 30 weeks pregnant. Mother was given combivir and nevirapine and her viral load fell from 20,000 copies/ml to 290 copies/ml at delivery. The infant was given zidovudine for 4 weeks after delivery. However pro-viral DNA for HIV was positive at birth in the infant, implying antenatal infection with HIV. The infant remained well, thrived and had a CD4 count above 35%. She was therefore not started immediately on antiretroviral therapy, but was given prophylactic co-trimoxazole.

On presenting with fever aged six months there were no physical signs of note. Blood cultures were taken which grew *Streptococcus pneumoniae *with reduced sensitivity to penicillin, resistant to trimethoprim but sensitive to cephalosporins and macrolides. She was given intravenous cefotaxime for 3 days then oral erythromycin for 2 weeks.

On review one month later she had been intermittently febrile since stopping antibiotics. Repeat blood cultures showed no growth, but she was given 14 days of parenteral ceftriaxone empirically. Following this she was commenced on combination antiretroviral therapy (cART; zidovudine, lamivudine, abacavir and nevirapine), because of these symptoms and falling CD4 count (17%{221 × 10^6^/l}).

She re- presented aged 8 months (one month after starting cART) with a swollen left wrist, fever and raised C-reactive protein (86 mg/l). A radio-isotope bone scan was compatible with osteomyelitis of the left radius. Blood cultures grew *Streptococcus pneumoniae *9S resistant to penicillin (MIC >2 mg/l) and trimethoprim, with reduced sensitivity to cephalosporins (MIC 1–2 mg/l), but sensitive to vancomycin, rifampicin and macrolides.

Aspiration of bone was not done since blood cultures were positive. She was given parenteral teicoplanin (10 mg/kg once daily) with oral rifampicin (10 mg/kg twice daily) and azithromycin (10 mg/kg once daily). After 2 weeks of treatment she developed rash and fever. These were thought to be a drug eruption and resolved when teicoplanin was stopped. She completed a 3 month course of rifampicin and azithromycin and continued on cART. Subsequent blood cultures were negative.

Eighteen months after treatment her left wrist is normal, she remains on her original cART regimen with an undetectable viral load and CD4 count of 34% (1398 × 10^6^/l).

## Discussion

*S pneumoniae *causes 3–6% of bone and joint infections in children with normal immunity [2,3]. Penicillin and/or ampicillin are the drugs most commonly used to treat bone and joint infections due to susceptible strains, whilst vancomycin and clindamycin or ceftriaxone are used for penicllin-nonsusceptible strains [3]. The best treatment for antibiotic resistant pneumococcal bone and joint infection is not known. Clinical response to treatment was similar in children with bone and joint infection due to penicillin non-susceptible pneumococci when compared with children infected by penicillin susceptible strains [2].

The combination of rifampicin and azithromycin was effective in treating osteomyelitis caused by a penicillin and cephalosporin non-susceptible pneumococcus in a child with HIV. Rifampicin and azithromycin have different sites of action in bacteria. This combination has been shown to be effective in animal models of staphylococcal osteomyelitis [4]. There is also one case report of an adult with antibiotic resistant pneumococcal infection in a prosthetic joint where these antibiotics were successfully used sequentially, but not in combination [5].

Both azithromycin and rifampicin achieve high levels in bone. However azithromycin monotherapy is ineffective for the treatment of experimental staphylococcal osteomyelitis, [6] whilst rifampicin monotherapy is associated with the emergence of rifampicin resistance.

In the UK macrolide resistance is more common in penicillin non-susceptible *S pneumoniae*, making macrolides in general less useful for treating osteomyelitis. However in this case the isolate was macrolide sensitive, despite penicillin and cephalosporin resistance.

Rifampicin can also interact with anti-retroviral drugs, especially protease inhibitors and nevirapine. Current advice is that low dose ritonavir/protease inhibitors combinations or nevirapine should not be given with rifampicin [7]. In our case we chose to continue nevirapine since we were unable to use efavirenz, due to the child's young age (less than 3 years), and felt there was no advantage in changing to a protease inhibitor. The child's viral load became undetectable on cART, despite the use of rifampicin.

Other antibiotics may have been effective in this case, but also had potential drawbacks. Ceftriaxone may be effective for non-ceftriaxone susceptible pneumococcal infection outside the central nervous system, as long as the MIC is < 2.0 mg/l [8]. However this reported series did not include any children with osteomyelitis. We felt we could not rely on this agent in an immunocompromised child with osteomyelitis, particularly since she had recently received a two week course of ceftriaxone and failed to respond clinically. Vancomycin is suggested for cephalosporin insensitive pneumococcal meningitis [9], but may have decreased effectiveness in bone and is unsuitable for outpatient therapy. We were unable to continue teicoplanin because of a drug eruption. The two weeks of teicoplanin our patient received was unlikely to have been sufficient treatment. Clindamycin is active in animal models of staphylococcal osteomyelitis [6]. However the suspension has a very poor taste, making it inappropriate in young children for the prolonged oral administration needed to treat osteomyelitis. Linezolid is active against pneumococci, and may have been effective [9]. However long term use can produce bone marrow depression. This would have been our choice if the child had failed to respond to azithromycin and rifampicin.

Preventive strategies against pneumococcal disease may have been useful in this child. Prophylactic co-trimoxazole gives some protection against bacterial infection in HIV positive children, but the organism in this case was trimethoprim resistant. Conjugate pneumococcal vaccine provides some protection, even in HIV positive children [10]. A 7 valent conjugate pneumococcal vaccine is now recommended for all HIV positive children in the UK. This may have given some protection against the pneumococcal serotype in this case by cross protection from the 9F component of the vaccine.

## Conclusion

The combination of rifampicin and azithromycin was well tolerated, simple to administer and effective in treating penicillin and cephalosporin insensitive pneumococcal osteomyelitis. This combination deserves further study for treatment of bone and joint infections, especially those due to antibiotic resistant Gram positive bacteria.

## Consent

Written informed consent was obtained from the patient's mother for publication of this case report and any accompanying images. A copy of the written consent is available for review by the Editor-in-Chief of this journal.

## Competing interests

The authors declare that they have no competing interests

## Authors' contributions

AR helped to draft and revised the manuscript. SA drafted the manuscript, CG revised the manuscript. All authors read and approved the final manuscript.
